# High-resolution magnetic resonance vessel wall imaging provides new insights into Moyamoya disease

**DOI:** 10.3389/fnins.2024.1375645

**Published:** 2024-04-11

**Authors:** Hui Yang, Guilan Huang, Xi Li, Moxin Wu, Weixin Zhou, Xiaoping Yin, Manqing Zhang, Zhiying Chen

**Affiliations:** ^1^Department of Neurology, Clinical Medical School of Jiujiang University, Jiujiang, China; ^2^Jiujiang Clinical Precision Medicine Research Center, Jiujiang, China; ^3^School of Basic Medicine, Jiujiang University, Jiujiang, China; ^4^Department of Neurology, University of California Irvine Medical Center, Irvine, CA, United States

**Keywords:** Moyamoya disease, cerebral artery occlusion, moyamoya vasculopathy, high-resolution magnetic resonance vessel wall imaging, intracranial vessel wall

## Abstract

Moyamoya disease (MMD) is a rare condition that affects the blood vessels of the central nervous system. This cerebrovascular disease is characterized by progressive narrowing and blockage of the internal carotid, middle cerebral, and anterior cerebral arteries, which results in the formation of a compensatory fragile vascular network. Currently, digital subtraction angiography (DSA) is considered the gold standard in diagnosing MMD. However, this diagnostic technique is invasive and may not be suitable for all patients. Hence, non-invasive imaging methods such as computed tomography angiography (CTA) and magnetic resonance angiography (MRA) are often used. However, these methods may have less reliable diagnostic results. Therefore, High-Resolution Magnetic Resonance Vessel Wall Imaging (HR-VWI) has emerged as the most accurate method for observing and analyzing arterial wall structure. It enhances the resolution of arterial walls and enables quantitative and qualitative analysis of plaque, facilitating the identification of atherosclerotic lesions, vascular entrapment, myofibrillar dysplasia, moyamoya vasculopathy, and other related conditions. Consequently, HR-VWI provides a new and more reliable evaluation criterion for diagnosing vascular lesions in patients with Moyamoya disease.

## 1 Introduction

Moyamoya disease (MMD) is a rare cerebral artery disease most commonly found in East Asian populations, with an annual incidence of 0. 94–4. 3 per 100,000 (Zhang et al., 2022). The typical features of MMD are the Internal carotid artery (ICA), middle cerebral artery (MCA), and anterior cerebral artery (ACA) progressive stenosis or occlusion, along with a collateral network of compensatory fragile vessels forming nearby (Kuroda et al., 2022). There are also studies claiming that these vessels of collateral circulation are actively formed (Kim et al., 2014) and that they appear “smoke-like” (called moyamoya in Japanese) in contrast (Suzuki and Takaku, 1969). The 2021 Moyamoya Research Committee’s updated criteria have revised the radiological diagnosis to specify that both bilateral and unilateral cases can be diagnosed as MMD (Kuroda et al., 2022). The diagnostic criteria for vascular disease range from DSA to MRI + MRA to HR-VWI. However, HR-VWI technology was gradually used in clinical practice after the discovery of black blood sequences to observe the structures on the vascular wall (Alexander et al., 2016; Mossa-Basha et al., 2016; Yuan and Parker, 2016; Mandell et al., 2017). For human tissues, HR-VWI is not limited to blood vessels but has been applied in bone, liver, and some other tissues (Kijowski and Fritz, 2023).

The latest research progress of HR-VWI in MMD shows that it is superior to other examination methods in MMD diagnosis, disease course detection, treatment, and prognosis evaluation (Das et al., 2023; Lu et al., 2023b; Yu et al., 2023). At present, the clinical examination items (DSA/CTA or MRA) can only detect the degree of lumen stenosis, but cannot distinguish the disease type. HR-VWI can present corresponding imaging characteristics in intracranial atherosclerosis (ICAD) (Ya et al., 2020), arterial dissection (Lee et al., 2021), vasculitis (Pfefferkorn et al., 2011; Edjlali et al., 2020), and MMD (Ya et al., 2020). HR-VWI can be used for these intracranial vascular diseases with vascular stenosis due to different etiologies.

## 2 Evaluation of cerebrovascular disease

Cerebrovascular diseases are evaluated based on morphology, structure, and function. The traditional way of evaluating morphology is by assessing the degree of blood vessel lumen stenosis, expressed as a percentage of the total vascular lumen, to determine disease severity and progression (Lehman et al., 2019; Ihara et al., 2022). However, there are many limitations in evaluating a single degree of stenosis (van den Wijngaard et al., 2016). In addition to reduced tube diameter, stenosis length is also associated with disease severity and progression (Mori et al., 1998). The structural evaluation assesses vascular lumen structure, and HR-VWI evaluates lumen and wall structure effectively. Luminal morphologic examination (DSA/CTA or MRA) may show normal stenosis in ischemic stroke patients. CT perfusion imaging is used for functional evaluation. Evaluating the blood supply capacity of blood vessels is crucial in ischemic stroke as stenosis or plaque rupture restricts blood flow causing cerebral infarction (Drouet, 2002). It is also necessary to evaluate the blood supply ability of blood vessels.

## 3 Pathologic features of MMD

**Figure 1:**
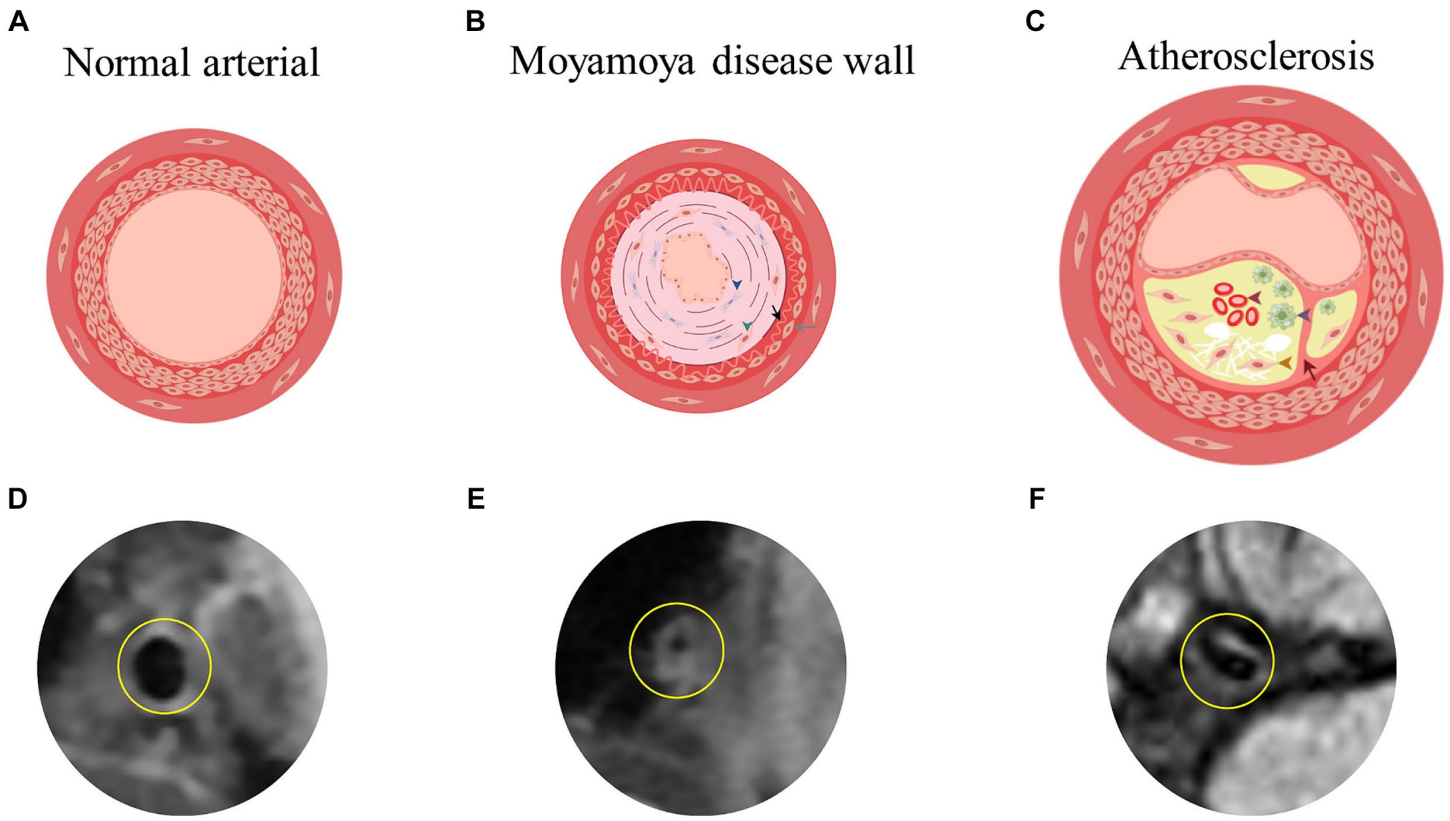
**(A,D)** Showed the simplified image of the three-layer membrane of the normal arterial wall and the actual HR-VWI of the normal artery wall. **(B)** Showed the damaged arterial wall in MMD, characterized by enhancement and bending of the intima and thinning of the media (blue triangles for fibroblast proliferation, green triangles for smooth muscle cell displacement and proliferation, black arrows for extremely curved and thickened intima, and gray arrows for fewer smooth muscle cells in the media). **(C)** Showed the atherosclerotic arterial wall, including subcutaneous lipid deposition, foam cell formation (purple triangle), smooth muscle cell displacement and proliferation (yellow triangle), and internal bleeding (red triangle) as well as rupture of the inner elastic membrane (red arrow). By Figdraw. **(E,F)** Showed the actual HR-VWI of patients with MMD or atherosclerosis. HR-VWI, High-Resolution Magnetic Resonance Vessel Wall Imaging; MMD, moyamoya disease.

MMD has six stages according to Japanese experts Suzuki et al. These stages are based on the degree of Moyamoya vasculopathy (MMV) expansion and the severity of stenosis or occlusion of major cerebral arteries as seen through MMD cerebrovascular angiography (Suzuki and Takaku, 1969). The lateral branch compensatory network develops as MMD progresses through these stages with collateral vessels such as basilar, choroidal, pericallosal, sigmoidal, and epidural (Pilgram-Pastor et al., 2022). Significant differences exist in the clinical manifestations of MMD in children and adults. MMD in children shows as recurrent transient ischemic attack (TIA) or headache (Tho-Calvi et al., 2018). Cerebral infarction and intellectual disability may occur after disease progression (Fukui et al., 2000). In adults, intracerebral, intraventricular, or subarachnoid hemorrhage often occurs. Severe disability and death may occur after intracerebral hemorrhage (ICH) (Fukui et al., 2000). In MMD patients, stenotic or occluded arteries show histopathologic features such as arterial smooth muscle cell proliferation, degeneration or death, intima-media thinning, elastic lamina fragmentation, and necrotic cellular component accumulation. This causes intimal enhancement and luminal narrowing (Guo et al., 2009). Autopsy studies show vacuolar degeneration of cerebrovascular smooth muscle cells (Lin et al., 2012). There is no evidence of significant inflammatory cell infiltration in the stenotic arteries (Fukui et al., 2000), however abnormal immune-related proteins are present (Lin et al., 2012). In contrast to ICAD, there was no significant inflammatory cell infiltration in the stenotic arteries of MMD (Zhang et al., 2019). In ICAD, the main cause of arterial stenosis is the formation of atherosclerotic plaques, which are composed mainly of a fibrous cap, foam cells cholesterol crystals, and foci of calcification (Cury et al., 2006) (Figure 1). Autoimmune diseases such as Graves disease and antiphospholipid syndrome have a close association with the progression of MMD (Chen et al., 2015; Mikami et al., 2019; Fox et al., 2021; Asselman et al., 2022). In a study by Li et al., hyperthyroidism was found in 0.9% of normal children, which is lower than the 10.5% seen in children with MMD (*p* = 0.003) (Li et al., 2011). In MMD, the ring finger protein 213 p.R4810K variant is strongly associated with vessel wall enhancement (VWE) and poor prognosis (Hao et al., 2024). A total of 16 million Asians living outside of Asia carry this RNF213 variant (Liu et al., 2012). Some studies have reported an independent association between the variant and recurrent stroke (Kim et al., 2020). In East Asia, MMD is tenfold more common than in European countries and the United States, at least in part because of variants (Koizumi et al., 2016). Additionally, it has been linked to a wide range of cardiovascular diseases (Okazaki et al., 2019).

## 4 Overview of HR-VWI

**Figure 2:**
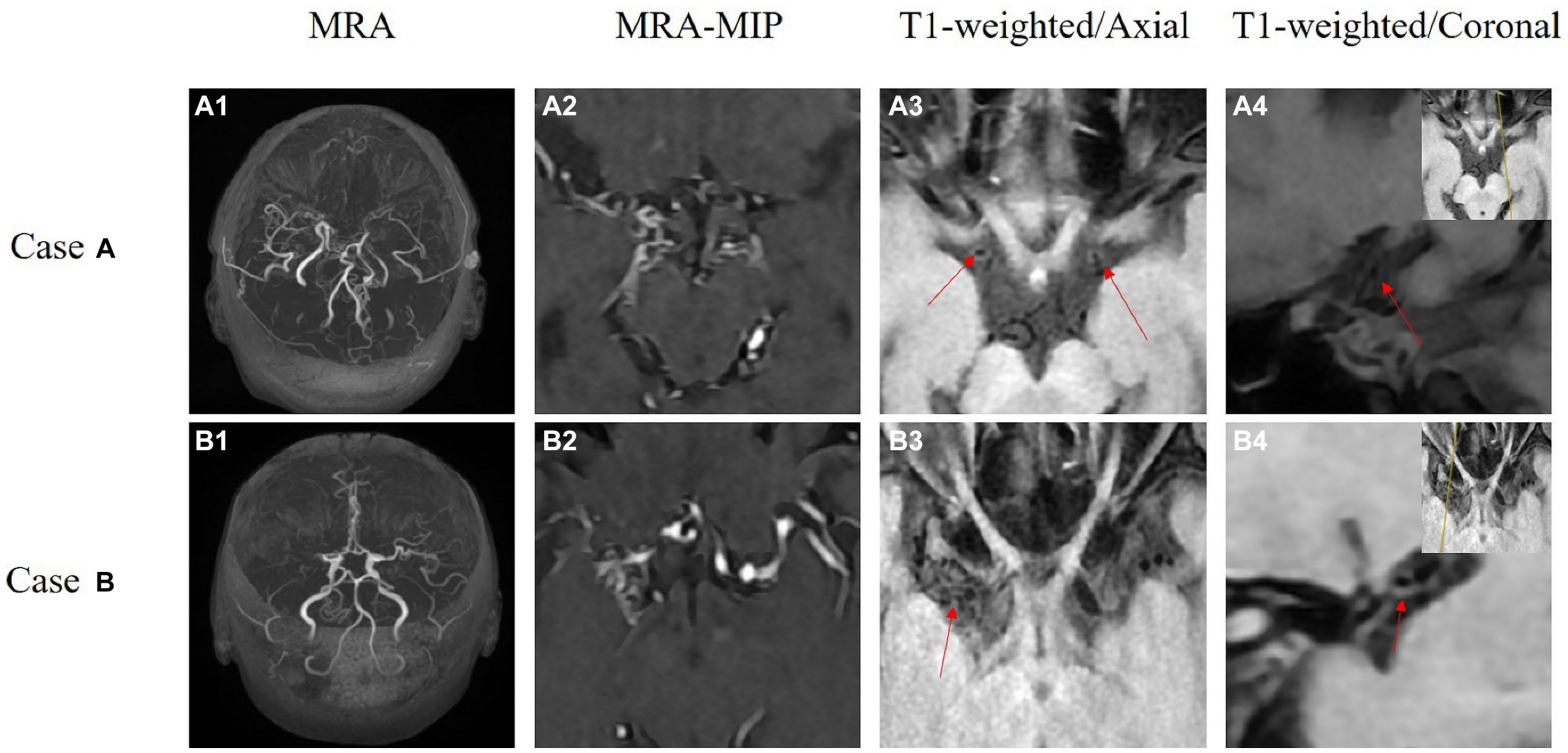
These are the HR-VWI of two MMD patients acquired using a 3.0 T MR scanner. Case A depicts bilateral MMD. **A1,A2** show revealing occlusion of bilateral internal carotid arteries to the middle cerebral arteries. **A3,A4** are high-resolution thin-slice scans of the middle cerebral arteries, where the red arrows indicate circular high signals. Case B is a patient with unilateral MMD. **B1,B2** show occlusion of the right middle cerebral artery. **B3,B4** present high-resolution thin-slice scans of the right middle cerebral artery, where the red arrows indicate circular high signals.

HR-VWI is an imaging technique that can visualize vessel lumen and detect pathological changes in arterial walls. It can identify intra-plaque lipids, calcification, and fibrous cap rupture in atherosclerotic plaques. HR-VWI is also more accurate than traditional angiographic methods (DSA/CTA or MRA) in detecting fresh hemorrhage (Yuan et al., 2001; Koo, 2015; Yu et al., 2015; Mossa-Basha et al., 2017; Young et al., 2019). HR-VWI requires a high-field-intensity nuclear magnetic resonance instrument and a high-resolution imaging coil. 3.0 T MR is widely used for detecting intracranial arterial blood walls (Li et al., 2014). At present, 7.0 T MR has been gradually applied to *in vivo* imaging, and there have been studies on imaging intracranial artery walls using 7.0 T MR (Deng et al., 2016). 7.0 T MR is used for *in vivo* imaging, but it may not help determine the Suzuki stage, ivy sign, and ICA diameter measurement (Deng et al., 2016; Dengler et al., 2016; Oh et al., 2017). However, further studies are needed to confirm this. Higher magnetic field strength leads to better image resolution or SNR. For 3.0 T MR head imaging, there are several receiving coils, including 8, 16, and 32-channel head coils, or 16, 20, 24, and 64-channel high-resolution coils for imaging the head and neck. Presently, the 32-channel high-resolution coil is commonly used (Kim et al., 2022; Liang et al., 2023). Studies have shown that, compared with 8-the channel head coil, the 32-channel head coil can increase SNR up to 3.4 times in the cortex and 1.4 times in the corpus callosum (Wiggins et al., 2006); Compared with the 12-channel head coil, the 32-channel head coil increased the SNR in the middle of the brain by about 1.5 times (Wiggins et al., 2006). For combined imaging of intracranial and extracranial arterial walls, a special head and neck joint coil is required (Hu et al., 2017). All of the above coils can only fit closely with adults, and then some scholars studied the coil that can fit closely with the head of children, and compared with the 32-channel coil sold in the market, the average SNR was 2.7 times higher (Ghotra et al., 2021) (Figure 2).

The core parameters of HR-VWI include imaging dimension, spatial resolution, and imaging sequence. The imaging dimensions include 2D and 3D (Mandell et al., 2017). The advantage of 3D imaging is that it can cover a larger imaging volume including the whole brain (Dieleman et al., 2014); 3D imaging can also be valuable for observing the morphology and distribution characteristics of intracranial arterial lesions from multiple angles (Mandell et al., 2017). Important reference for endovascular treatment such as stent implantation (Lou et al., 2014). At the same time, a large amount of image data is generated, which brings certain challenges to the analysis (Li et al., 2019). For 3D vessel wall imaging of intracranial arteries at spatial resolution, an anisotropic resolution of ≤0. 5 mm × 0. 5 mm × 0. 5 mm is recommended (Li et al., 2019). Enhance with 3D imaging, as CSF pulsation artifacts are evident as well as flow-related signals. Imaging sequences using blood and cerebrospinal fluid signal suppression sequences are commonly used today, including spin echo, double inversion recovery (DIR), and fast spin echo sequences. DIR is useful for thinner tissue layers due to its ability to suppress blood flow signals. Other techniques include spatial pre-saturation (saturation band), motion-sensitized driven-equilibrium pre-pulse (MSDE), and delay alternating with nutation for tailored excitation (DANTE) (Li et al., 2019). MSDE and DANTE can be used for thicker layers (of tissue), while DANTE is currently limited to experimental research (Li et al., 2014). The variable-angle fast spin echo sequence (VFAFSE/TSE) obtained by improvement is currently the main acquisition sequence for intracranial 3D vascular wall imaging, with advantages such as high intra-layer resolution, high SNR, and insensitivity to magnetic field inhomogeneity (Li et al., 2019). Based on the above advantages, VFAFSE/TSE has been widely used for arterial wall imaging of intracranial arteries (Huang et al., 2023), carotid arteries (Saba et al., 2023), aorta (Hu et al., 2020), and thoracic great vessels (Treitl et al., 2017). Image contrast is also included in the imaging sequence, which requires the sum of image information with multiple contrasts, mainly including the time-leap method, PDWI, T1WI, and T2WI. Among them, T1WI can fully display the location, morphology, reconstruction, and signal characteristics of intracranial arterial lesions (Dieleman et al., 2014) (such as whether the vascular wall is enhanced or not) (Lu et al., 2021). Some scholars have found that the high T2WI signal of the intracranial artery wall helps identify the nature of vascular wall lesions (Mossa-Basha et al., 2017), and can also be used to evaluate the external diameter of the vascular lesions (Kuroda et al., 2015), but further verification is needed.

## 5 Application of HR-VWI in MMD

**Figure 3:**
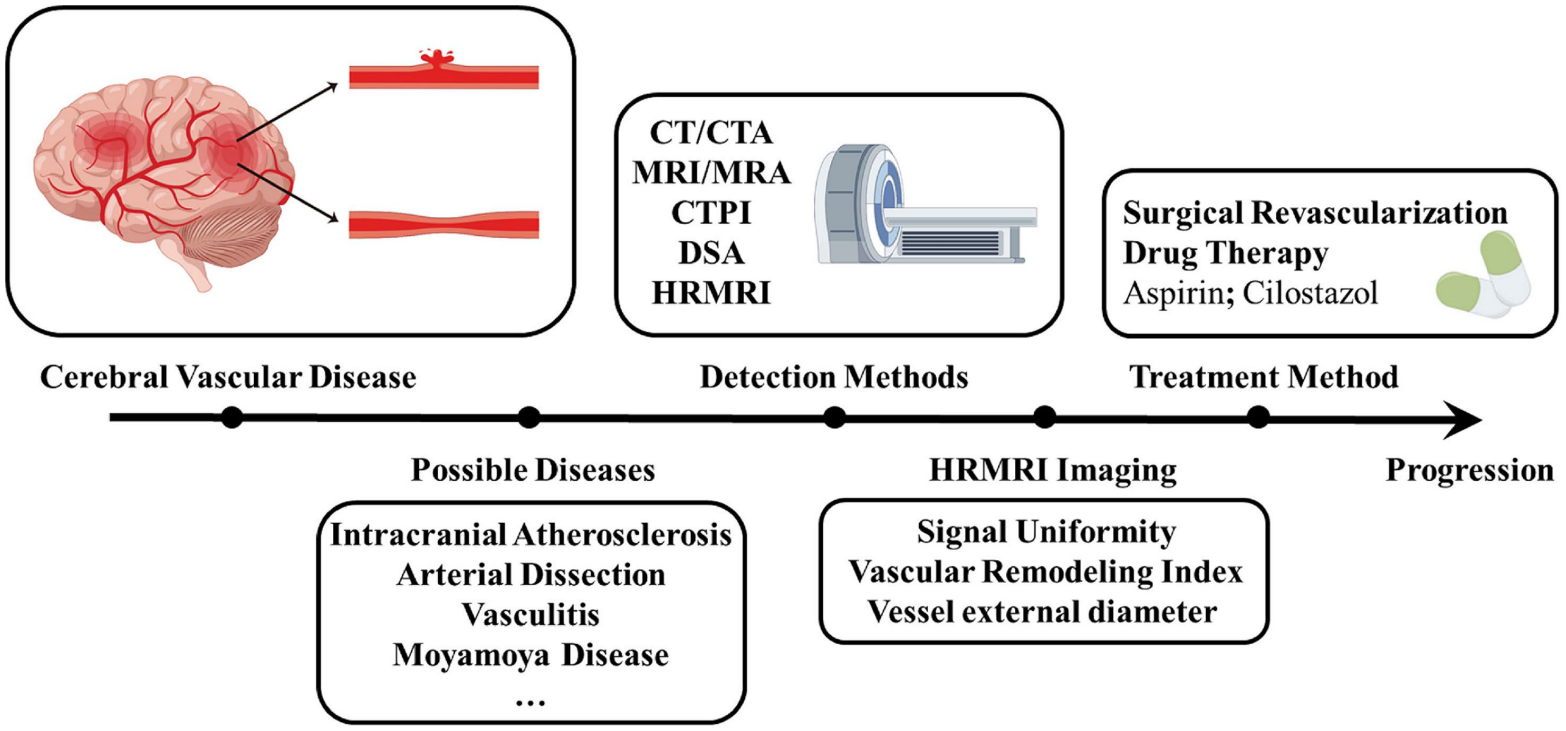
Progression of diagnosis and treatment of Moyamoya disease. Shows the process of diagnosing and treating a patient with a cerebrovascular disease that is eventually diagnosed as a Moyamoya disease after multiple detection methods have been performed. By figdraw. CTA, Computed tomography angiography; MRA, Magnetic resonance angiography; CTPI, Computed tomography perfusion imaging; DSA, Digital subtraction angiography; HR-VWI, High-Resolution Magnetic Resonance Vessel Wall Imaging.

A patient was diagnosed with MMD when at least two of the following three HR-VWI features were found in the affected segment of the ICA terminal segment or the MCA/ACA proximal segment: (1): No eccentric wall enhancement (Wu et al., 2021), (2): Vessel external diameter reduction (Kuroda et al., 2022), (3): uniform signal (Yang et al., 2021; Lu et al., 2023b). Eccentric wall enhancement refers to eccentric or focal wall enhancement evident on HR-VWI. The thickest part of the wall is estimated to be twice as thick as the thinnest part of the vascular lesion (Wang et al., 2017). The eccentricity index is calculated as follows: eccentricity index = (maximum vessel wall thickness − minimum vessel wall thickness) / maximum vessel wall thickness. If the eccentricity index ≥0. 5, it is considered eccentric VWE, otherwise, it is centripetal VWE (Shishikura et al., 2020). When the outer diameter of the involved artery is significantly smaller than the normal adjacent distal site or normal reference value, it indicates that the outer diameter is reduced (Kaku et al., 2012). T2WI sequences were used to assess the outer diameter of stenotic vessels at the site of minimal outer diameter (Kuroda et al., 2015). Signal heterogeneity was considered when stenotic vessel signal intensity was found to include a mixture of signals with high or low signal intensity in T1WI sequences; otherwise, signal homogeneity was considered (Lu et al., 2021). Using the above HR-VWI diagnostic criteria can improve the diagnostic rate of MMD (Figure 3).

### 5.1 Role of HR-VWI in the diagnosis of MMD

VWE plays a crucial role in diagnosing and monitoring MMD through HR-VWI. Studies have found that HR-VWI of MMD patients mostly have the following characteristics: concentric stenosis appears in the vessel wall at the distal ICA or the beginning of MCA, with uniform signals (Yuan et al., 2015) and VWE indicating the progression of MMD vascular lesions (Roder et al., 2019). Some studies use the vascular remodeling index to perform HR-VWI evaluation on the stenosis characteristics of MMV. In MMD patients, vascular stenosis is mainly due to negative remodeling. The VWE degree in MMD is lower than in ICAD-induced stenosis (*p* < 0.01) (Ya et al., 2020). HR-VWI studies show that MCA wall signal uniformity is higher in MMD patients (85.7%) than in ICAD patients (32.6%) (*p* < 0.001) (Yuan et al., 2015). ICAD-induced stenosis is usually characterized by eccentric and inhomogeneous enhancement of the vessel wall (Ryoo et al., 2014). Researchers evaluated MCA using HR-VWI with 3 vessel wall characteristics to differentiate between MMD and atherosclerosis-associated moyamoya vasculopathy (AS-MMV). Patients with AS-MMV had higher outer diameter, remodeling index, and pattern of VWE (all *p* < 0.001). The outer diameter had a better diagnostic performance compared to the remodeling index in distinguishing AS-MMV from MMD (outer diameter vs. remodeling index: 0.912 vs. 0.889, *p* = 0.007) (Liu et al., 2023). MMD causes the narrowing of cerebral arteries and leads to the development of side branches known as MMV. This can result in hemorrhage with a high risk of recurrence and rebleeding in hemorrhagic MMD (Funaki et al., 2019; Wang et al., 2020). This is because the pathophysiologic changes in MMD usually include the formation of abnormal collateral vessels caused by perforating arteries (Takagi et al., 2007). Branching extensions of perforating arteries can form abnormal connections known as collateral pathways. These connections increase vascular fragility and lead to microaneurysms, which can cause intracranial hemorrhage (Yamashita et al., 1983; Matsushige et al., 2016; Rhim et al., 2018). T1-weighted black blood HR-VWI shows perforating arteries in MMD patients clearly, while 3.0 T MR HR-VWI characterizes their morphological changes (Okuchi et al., 2014; Wu et al., 2021). This also suggests that identifying the side branches also helps to diagnose MMD.

### 5.2 Role of HR-VWI in monitoring the progress of MMD

HR-VWI can provide detailed information about the anatomy of MMD and the abnormal vascular network, which can help assess the risk of ischemia or hemorrhage in MMD (Lu et al., 2021; Zhao et al., 2021). One study studied 29 patients with MMD using 3.0 T HR-VWI intracranial vessel wall imaging and found that VWE was positively correlated with ischemic events (Kathuveetil et al., 2020). Another study showed that VWE could be used as a predictor of arterial stenosis and poor prognosis in MMD (Lu et al., 2023a). However, some studies claim that lumen and vessel outer wall diameters decrease with disease severity, but there is no significant difference in wall thickness (Cogswell et al., 2020). Some scholars have used HR-VWI to analyze symptomatic isolated MCA steno-occlusive disease (MCAD) that is not caused by atherosclerosis and found that MMD is the cause of some non-atherosclerotic lesions. Therefore, symptomatic isolated MCAD may be an early manifestation of MMD in young Asian people (Kim et al., 2016). Another study explored the correlation between choroidal anastomosis and bleeding events in 50 adult patients with advanced MMD using HR-VWI and found that choroidal anastomosis was significantly correlated with hemorrhage (Hirano et al., 2020; Wu et al., 2021). MMD patients with anterior ICH have also been analyzed, and it has been found that the coexistence of reduced lenticulostriate artery and relatively preserved lumen area of the distal ICA may be a potential predictor of anterior ICH in patients with MMD (Xu et al., 2022). In investigating the clinical significance of periventricular collateral enhancement sign (PCES) in adult patients with MMD through vascular wall imaging, it was found that PCES was the only independent factor in bleeding in patients with MMD. It can be seen that PCES can help detect major blood vessels that cause bleeding in MMD patients (Ryu et al., 2022). Using HR-VWI to predict stroke incidence in conservatively treated MMD patients, one prospective study concluded that VWE was significantly associated with an increased incidence of stroke (HR, 4.16; 95% CI, 1.55–11.15) (Zhang H. et al., 2023). However, the small number of subjects in most studies may have biased the final results. It is important to include more patients with MMD in future studies of MMD course progression.

### 5.3 Role of HR-VWI in the evaluation of MMD treatment

At present, surgical revascularization is the main treatment for MMD patients (Bao and Duan, 2023), however, surgical treatment may lead to perioperative complications such as ischemia or hydrocephalus (Fujimura et al., 2011). With a better understanding of the pathophysiology of MMD, non-surgical approaches targeting the pathogenesis of MMD can be used to prevent or slow the progression of the disease (Chen T. et al., 2023). In a 2018 national study in Japan, it was noted that the mechanism by which an antiplatelet agent (APT) is used to treat MMD is unknown, but it was summarized that aspirin, cilostazol, and clopidogrel are used in the course of MMD treatment, with aspirin being the most commonly used (Oki et al., 2018). An analysis of 14 years of follow-up showed that APT treatment (Aspirin HR, 0.77; 95% CI, 0.61–0.97, *p* = 0. 03; Clopidogrel HR, 1.08; 95% CI, 0.97–1.21, *p* = 0.15) can significantly improve the survival rate of MMD patients, especially cilostazol (HR, 0.64; 95% CI, 0.54–0.75, *p* < 0. 01), which had greater survival benefits (Seo et al., 2021). In a retrospective study in 2023, it was found that APT could reduce the mortality of patients with hemorrhagic MMD and did not increase the risk of hemorrhagic stroke, all of which indicated that APT may benefit MMD patients (Luo et al., 2023). At the same time, some studies have suggested that perfusion improvement in MMD patients, rather than antiplatelet and statins, maybe the main step in decreasing MMD-associated strokes (Chen et al., 2022). Subsequent studies using HR-VWI were able to demonstrate the specific mechanism played by cilostazol in patients with MMD. It was demonstrated that cilostazol resulted in a significant increase in the outer diameter of the diseased vessel, a significant reduction in the degree of stenosis, and positive remodeling (Kim et al., 2022). This is also the only study that used HR-VWI to analyze medical interventions for MMD. According to the analysis results, HR-VWI is a better technique to investigate the specific effects of therapeutic drugs on patients with MMD (Table 1).

**Table 1:** The application of HR-VWI in MMD.

Article	Author’s name	Application	Year	References
Long-term outcomes of moyamoya disease versus atherosclerosis-associated moyamoya vasculopathy using high-resolution MR vessel wall imaging	Lu et al.	Comparison of long-term outcomes and surgical advantages of MMD and AS-MMV using high-resolution magnetic resonance imaging (HRMRI).	2023	Lu et al. (2023b)
High-resolution vessel wall magnetic resonance imaging in intracranial vasculopathies: an experience from eastern India	Das et al.	Adjunctive use of HR-VWI, in clinical and luminal assessment, can significantly improve diagnostic accuracy during the evaluation of intracranial vasculopathies, with its greatest utility in diagnosing ICAD, CNS angiitis, and dissection.	2023	Das et al. (2023)
High-resolution combined arterial spin labeling MR for identifying cerebral arterial stenosis induced by moyamoya disease or atherosclerosis.	Ya et al.	Differentiating MMD from intracranial atherosclerotic stenosis (IAS).	2020	Ya et al. (2020)
High-resolution MRI vessel wall enhancement in moyamoya disease: risk factors and clinical outcomes	Hao et al.	Intracranial VWE on HRMRI is associated with the progression and poor prognosis of MMD.	2024	Hao et al. (2024)
Vessel Wall Changes on Serial High-Resolution MRI and the Use of Cilostazol in Patients With Adult-Onset Moyamoya Disease	Kim et al.	Serial High-Resolution Magnetic Resonance Imaging to Measure Changes in Outer Diameter, Luminal Stenosis, and Vascular Enhancement in MMD Patients Receiving Platelet Therapy.	2022	Kim et al. (2022)
Association of intracranial vessel wall enhancement and cerebral hemorrhage in moyamoya disease: a high-resolution magnetic resonance imaging study	Lu et al.	To investigate the enhancement characteristics of the vascular wall and its relationship with initial and recurrent intracranial hemorrhage in patients with MMD using three-dimensional HRMRI.	2021	Lu et al. (2021)
Validation of choroidal anastomosis on high-resolution magnetic resonance imaging as an imaging biomarker in hemorrhagic moyamoya disease	Wu et al.	HRMRI can provide detailed information on both the anatomies and abnormal collaterals in MMD, which facilitates risk estimates of bleeding in MMD.	2021	Wu et al. (2021)
High-resolution MRI of the vessel wall helps to distinguish moyamoya disease from atherosclerotic moyamoya syndrome	Yang et al.	To evaluate the value of high-resolution magnetic resonance imaging of the vessel wall (VWI) for differentiating MMD from atherosclerotic moyamoya syndrome (AS-MMS).	2021	Yang et al. (2021)
The Contrast Enhancement of Intracranial Arterial Wall on High-resolution MRI and Its Clinical Relevance in Patients with Moyamoya Vasculopathy	Wang et al.	To investigate the characteristics of intracranial vessel wall strengthening in patients with MMV and its relationship to ischemic infarction.	2017	Wang et al. (2017)
The diagnostic performance of high-resolution magnetic resonance-vessel wall imaging in differentiating atherosclerosis-associated moyamoya vasculopathy from moyamoya disease	Liu et al.	HRMR-VWI is useful in distinguishing MMD from AS-MMV. The outer diameter of the MCA performs better in distinguishing AS-MMV from MMD than the remodeling index and wall enhancement patterns.	2023	Liu et al. (2023)
Characteristics of symptomatic plaque on high-resolution magnetic resonance imaging and its relationship with the occurrence and recurrence of ischemic stroke	Zhao et al.	To determine the characteristics of asymptomatic plaques in patients with intracranial atherosclerosis demonstrated by HRMRI and their association with the occurrence and recurrence of ischemic stroke events.	2021	Zhao et al. (2021)
Vessel Wall Thickening and Enhancement in High-Resolution Intracranial Vessel Wall Imaging: A Predictor of Future Ischemic Events in Moyamoya Disease	Kathuveetil et al.	To study the high-resolution imaging features of MMD and their correlation with recent ischemic events.	2020	Kathuveetil et al. (2020)
Vessel wall enhancement as a predictor of arterial stenosis progression and poor outcomes in moyamoya disease	Lu et al.	To determine the relationship between VWE and progression of arterial stenosis and the clinical prognosis of patients with MMD using HR-VWI.	2023	Lu et al. (2023a)
Vessel Wall and Lumen Features in North American Moyamoya Patients	Cogswell et al.	To apply intracranial VWI to determine changes in vessel wall characteristics between North American moyamoya patients and controls, as well as with standard clinical measures of moyamoya disease severity.	2020	Cogswell et al. (2020)
Imaging features of adult moyamoya disease patients with anterior intracerebral hemorrhage based on high-resolution magnetic resonance imaging	Xu et al.	To determine the HRMRI features of patients with MMD accompanied by anterior ICH and to attempt to reveal the underlying mechanisms of anterior ICH.	2022	Xu et al. (2022)
Predictors of Stroke Outcomes in Conservatively Treated Patients With Moyamoya Disease: A Follow-up MRI Study	Zhang et al.	HR-VWI was utilized to predict the incidence of stroke in patients with multiple sclerosis undergoing conservative treatment, concluding that arterial wall enhancement, modified Rankin Scale scores ≥3, and reduced CBV were significantly associated with an increased incidence of stroke.	2023	Zhang H. et al. (2023)

HR-VWI has its advantages in MMD diagnosis, disease course monitoring, and treatment risk prediction. In the diagnosis of MMD, ICAD, and MMD are distinguished according to the characteristics of vascular wall imaging, and the characteristics of vascular imaging of MMD are summarized to provide a reference for clinical workers. In the latest research on MMD course monitoring, it can be seen that HR-VWI can more clearly investigate the related vascular lesions that may cause stroke events during MMD. In the process of treatment and prognosis of MMD, it can be used as a detection tool to timely detect the specific sites and effects of drugs or treatments in MMD, to adjust to the appropriate degree to achieve better therapeutic effects.

## 6 Comparison of HR-VWI with other imaging tools

Diagnostic evaluation of cerebrovascular disease includes transcranial Doppler (TCD), CTA, MRA, and DSA (Koo, 2015). DSA is currently the gold standard for the diagnosis of MMD but may capture overlapping imaging features and be more invasive (Park et al., 2017; Ihara et al., 2022; Waddle et al., 2022). A comparative analysis of studies has shown that HR-VWI improves diagnostic confidence in conditions where clinical or DSA imaging features are unclear (Park et al., 2017), and also identifies vascular remodeling, which may be clinically important but cannot be recognized by conventional imaging. TCD is a rapid, noninvasive diagnostic technique that determines the degree of stenosis based on the velocity of blood flow in the cerebral vasculature (Wan et al., 2022; Chen J. Y. et al., 2023). MRA can noninvasively identify the characteristic architecture of MMD and can intimate areas of major occlusions, reconstructions, and small-vessel collateral flow (Togao et al., 2018), but with limited resolution and poor display of fine arterioles (Li et al., 2019). HR-VWI has higher imaging clarity and precision and can visualize the subtle changes in the vessel wall. CTA scanning is fast, but requires injection of contrast medium and, a higher radiation dose, and the MMV display at the base of the skull is easily interfered with by the skull bone (Byworth et al., 2021). HR-VWI is non-invasive and radiation-free compared to the other detection methods. Given that conventional imaging techniques are more focused on the degree of luminal stenosis and are more limited, there is a tendency for misdiagnosis for a disease that manifests solely initially with luminal stenosis. This can lead to patients not being able to obtain the correct treatment in time, which further leads to a series of consequences including the decline in the survival period and overall poor prognosis (Table 2).

**Table 2:** Comparison of HR-VWI with other imaging tools.

	Advantages	Disadvantages
HR-VWI	Clear lumen structure Higher clarity Noninvasive No irradiation Predict future high-risk populations	Lack of harmonized image analysis standards Lack of pathologic controls and pathophysiologic analysis
DSA	Gold standard	Invasive Expensive Poor quantification degree of lumen stenosis
CTA	Higher scanning speed	Cranial disturbance High radiation Poor quantification degree of lumen stenosis
MRA	Noninvasive	Limited resolution Poor display Degree of lumen stenosis
TCD	Noninvasive Provide real-time hemodynamic data	Higher demands on the operating surgeon

HR-VWI, High-Resolution Magnetic Resonance Vessel Wall Imaging; DSA, Digital subtraction angiography; CTA, Computed tomography angiography; MRA, Magnetic resonance angiography; TCD, Transcranial Doppler.

## 7 Conclusion and prospect

HR-VWI for diagnosing MMD faces challenges such as the lack of unified scanning sequences, parameters, and standardized analysis guidelines. Long MRI acquisition time increases the risk of missing the optimal window for revascularization (Bao and Duan, 2023). HR-VWI application in MMD also lacks pathologic control and pathophysiologic analysis because it is difficult to obtain arterial specimens from living patients (Yu et al., 2015), so the study of specific tissue components of intracranial vessels is still limited. Advanced intracranial wall imaging techniques are being explored for better clinical application. Further studies are needed to validate HR-VWI features and determine its predictive power for treatment and stroke (Bodle et al., 2013; Wang et al., 2021). Use HR-VWI to observe various drug therapies beyond APT, such as drugs to increase angiogenesis, anticancer drugs to reduce smooth muscle cell proliferation, retinoids, and other specific changes in diseased blood vessels of MMD patients (Guo et al., 2009; Bang et al., 2015; Lee et al., 2015). Use HR-VWI technology to investigate MMD patients in different stages and explore potential pathogenesis. Combine emerging technologies, like AI, to improve HR-VWI performance (Kim et al., 2019). A Pseudo-Three-Dimensional Residual Network has been proposed to process spatio-temporal information and combined with DSA to recognize MMD (Xu et al., 2023), and a 3D coordinate attention residual network has also been used in combination with MRA to evaluate stenotic occlusion changes in MMD (Zhang Z. et al., 2023). In the future, it is possible to use these techniques in combination with HR-VWI. Compressed sensing techniques can be used to reduce redundant imaging data acquisition (Yamamoto et al., 2018). HR-VWI can be combined with other imaging techniques for clinical decision-making.

## Author contributions

HY: Data curation, Resources, Visualization, Writing – original draft. GH: Data curation, Writing – original draft. XL: Writing – original draft, Writing – review & editing. MW: Data curation, Writing – original draft. WZ: Writing – original draft. XY: Funding acquisition, Writing – original draft. MZ: Conceptualization, Writing – original draft, Writing – review & editing. ZC: Conceptualization, Funding acquisition, Writing – original draft, Writing – review & editing.

## Glossary

MMD: Moyamoya disease
HR-VWI: High-Resolution Magnetic Resonance Vessel Wall Imaging
ICA: Internal carotid artery
MCA: Middle cerebral artery
ACA: Anterior cerebral artery
ICAD: Intracranial atherosclerosis
DSA: Digital subtraction angiography
CTA: Computed tomography angiography
MRI: Magnetic resonance imaging
MRA: Magnetic resonance angiography
TIA: Transient ischemic attack
ICH: Intracerebral hemorrhage
MMV: Moyamoya vasculopathy
VWE: Vessel wall enhancement
SNR: Signal-to-Noise Ratio
DIR: Double inversion recovery
MSDE: Motion-sensitized driven-equilibrium
DANTE: Delay alternating with nutation for tailored excitation
MCAD: Middle cerebral artery steno-occlusive disease
PCES: Periventricular collateral enhancement sign
APT: Antiplatelet agent
TCD: Transcranial Doppler
